# Increased anterior insula activity in anxious individuals is linked to diminished perceived control

**DOI:** 10.1038/tp.2015.84

**Published:** 2015-06-30

**Authors:** R P Alvarez, N Kirlic, M Misaki, J Bodurka, J L Rhudy, M P Paulus, W C Drevets

**Affiliations:** 1 Laureate Institute for Brain Research, Tulsa, OK, USA; 2 Faculty of Community Medicine, The University of Tulsa, Tulsa, OK, USA; 3 Department of Psychology, The University of Tulsa, Tulsa, OK, USA; 4 Center for Biomedical Engineering, The University of Oklahoma, Norman, OK, USA; 5 College of Engineering, The University of Oklahoma, Norman, OK, USA; 6 Janssen Research and Development, Janssen Pharmaceuticals, Titusville, NJ, USA

## Abstract

Individuals with high-trait anxiety frequently report decreased perceived control. However, it is unclear how these processes are instantiated at a neural level. Prior research suggests that individuals prone to anxiety may have exaggerated activity in the anterior insula and altered activity in the cingulate cortex during anticipation of aversive events. Thus, we hypothesized that anxiety proneness influences anterior insula activation during anticipation of unpredictable threat through decreased perceived control. Forty physically healthy adults underwent neuroimaging while they explored computer-simulated contexts associated either with or without the threat of an unpredictable shock. Skin conductance, anxiety ratings and blood oxygenation level-dependent (BOLD) functional magnetic resonance imaging were used to assess responses to threat versus no threat. Perceived control was measured using the Anxiety Control Questionnaire-Revised. Mediation analysis examined how anxiety proneness influenced BOLD activity. Anticipation of unpredictable threat resulted in increased skin conductance responses, anxiety ratings and enhanced activation in bilateral insula, anterior midcingulate cortex (aMCC) and bed nucleus of the stria terminalis. Individuals with greater anxiety proneness and less perceived control showed greater activity in dorsal anterior insula (dAI). Perceived control mediated the relationship between anxiety proneness and dAI activity. Increased dAI activity was associated with increased activity in aMCC, which correlated with increased exploratory behavior. Results provide evidence that exaggerated insula activation during the threat of unpredictable shock is directly related to low perceived control in anxiety-prone individuals. Perceived control thus may constitute an important treatment target to modulate insula activity during anxious anticipation in anxiety-disordered individuals.

## Introduction

Trait anxiety, a predisposition to respond anxiously, has been linked to dysregulation of mood and anxiety,^1^ but the underlying brain processes by which anxiety proneness affects neural systems remain unclear. Perceived control over aversive events is an important psychological construct that links a specific cognitive schema to an experienced emotion,^2^ and may be a possible mediator between high-trait anxiety and exaggerated neural processing of emotionally significant events. Theories of psychopathology posit that a lack of perceived control is central to the etiology and maintenance of negative affect and emotional disorders.^3, 4^ Supporting this view, the results of a recent meta-analysis suggested that reduced perceptions of control are associated with greater trait anxiety in both nonclinical and clinical samples, particularly in healthy adults and in individuals with generalized anxiety disorder.^5^ In a related study, treatment-seeking individuals with generalized anxiety disorder, panic disorder, social phobia and obsessive-compulsive disorder who initiated cognitive-behavioral therapy, subsequently reported large increases in perceived control and greater reductions in anxiety symptoms compared with untreated individuals, suggesting that perceived control might predict recovery from some anxiety disorders following cognitive-behavioral therapy.^6^ Elucidating whether anxiety proneness affects neural systems indirectly through perceived control conceivably may facilitate the development of more effective interventions capable of enhancing perceptions of control and reducing the detrimental effects of diminished perceived control.

Functional neuroimaging data obtained in nonclinical subjects have suggested that insular cortex may be critically involved in the anticipation of aversive events, including negatively valenced pictures,^7, 8^ loud sounds,^9^ monetary loss,^10^ thermal stimuli,^11^ electric stimuli^12^ and aversive unconditioned stimuli predicted by either cue or context conditioned stimuli.^13, 14^ Although these prior reports suggest that the insula is broadly involved in anxious anticipation, studies that have compared anticipation of temporally predictable and unpredictable aversive events indicate that activity in anterior insula is heightened during anticipation of unpredictable aversive events compared with predictable aversive events.^15, 16^ In addition, studies that differentiated sustained from transient anticipatory processing found that activity in anterior insula is particularly enhanced during sustained versus transient anticipation of aversive events.^17, 18, 19^ These findings strongly suggest that anterior insula has an important role in the anticipation of aversive events, especially sustained anticipation of unpredictable aversive events.

Notably, evidence of altered insula activation is also emerging as a key finding in individuals with dysregulated mood or anxiety.^20, 21, 22, 23, 24^ For example, individuals with major depressive disorder show enhanced activity in right anterior insula during the anticipation of painful temperature stimuli compared with healthy controls.^25, 26^ Compared with non-traumatized controls, women with a history of intimate partner violence and consequent posttraumatic stress disorder show greater hemodynamic activity in right anterior insula during the anticipation of negative images.^27^ Similarly, compared with combat-exposed controls, combat veterans with posttraumatic stress disorder show increased activation in right anterior insula during the anticipation of negative combat-related images presented unpredictably.^28^ The magnitude of right anterior insula activity in response to unpredictably presented versus predictably presented images was associated with posttraumatic stress disorder severity, such that right anterior insula activity is heightened in combat veterans with more severe posttraumatic stress disorder. Moreover, in the combat-exposed healthy controls from this study, activity in right anterior insula was associated with greater perceived threat. Several studies also report that nonclinical subjects with high trait anxiety compared with those having low-trait anxiety show enhanced activation in anterior insula during emotional processing and the anticipation of aversive images.^29, 30, 31^ These results indicate that individuals with dysregulated mood or anxiety manifest altered activity in anterior insula during the anticipation of aversive events.

Studies that have compared individuals with high-trait and low-trait anxiety during decision making,^32^ or have examined anxious anticipation of threat, that is, aversive events,^7, 12, 15, 17, 18^ frequently report concomitant activation in the anterior insula and the supracallosal portion of the anterior cingulate gyrus referred to as the midcingulate cortex (MCC).^33, 34^ While much remains to be clarified about the functional relationships between these regions, their joint activation during the anticipation of threat is supported by the substantial neuroanatomical connections extant between anterior insula and the anterior MCC (aMCC).^35, 36^ These regions are emerging as key nodes in the ‘salience network', a brain system thought to have a central role in the detection of behaviorally relevant stimuli and the coordination of adaptive responses.^33, 37, 38^

Recent meta-analyses examining the functional organization of the insula indicate that anterior insula consists of important functional subdivisions in humans^39, 40, 41, 42^ consistent with anatomical studies in monkeys.^43^ Converging evidence from these studies suggest that anterior insula consists of a dorsal anterior subregion that is relatively more involved in cognitive processes and a ventral anterior subregion more involved in affective processes. On the basis of this research and the presumption that perceived control is primarily a cognitive process, we hypothesized that any influence of perceived control on insula activity would specifically involve dorsal anterior insula. We therefore examined whether anxiety proneness affects the anterior insula through diminished perceived control during anxious anticipation. We used blood oxygenation level-dependent (BOLD) functional magnetic resonance imaging (fMRI), electrodermal recording and anxiety ratings to explore the relationships between perceived control and the brain responses, skin conductance and subjective anxiety elicited during the anticipation of unpredictable threat (AUT). Using mediation analysis, we tested the hypothesis that anxiety proneness specifically influences BOLD activity in the dorsal anterior insula indirectly through perceived control. Correlation analyses were applied to examine the relationship between brain activation and behavioral measures, and to explore the functional relationship between dorsal anterior insula and MCC. Finally, because the bed nucleus of the stria terminalis (BNST) has been implicated in modulating sustained anxiety responses analogous to those elicited herein to a context in which an aversive event may occur that otherwise is unpredicted by any sensory cue,^17, 44^ and in preclinical and clinical studies of the functional anatomy of sustained apprehensive states,^45^ we also examined activity in the BNST. The main results suggest that the link between anxiety proneness and activity in the dorsal anterior insula is mediated by reduced perceptions of control.

## Materials and methods

### Participants

Forty physically healthy volunteers (37 right-handed, two left-handed, one ambidextrous) between the ages of 18 and 50 years were recruited (20 females; mean age=31 years, s.d.=10) to participate in fMRI while performing an ‘anticipation of unpredictable threat' task. All the subjects provided informed consent according to procedures approved by the Western Institutional Review Board, and underwent a Structured Clinical Interview for DSM-IV-TR Axis I Disorders (SCID-I) performed by a Master's-level clinician with experience and training in the administration of the SCID-I interview. Exclusion criteria included past or current psychiatric disorders as per SCID-I, major neurological or medical disorders, substance abuse, current pregnancy and exposure to psychotropic medications or other drugs likely to influence cerebral function or blood flow within 3 weeks. Subjects with a body mass index >35 kg /m^2^ also were excluded from participation to minimize difficulty obtaining electromyographic recordings during individual threshold testing before scanning. All the subjects received monetary compensation for their participation.

### Measures

Participants completed the STAI (State-Trait Anxiety Inventory), Form Y, a 40-item self-report measure of both trait anxiety (STAI-T) assessing individual differences in anxiety proneness, and state anxiety (STAI-S) assessing feelings of apprehension in response to current circumstances.^46^ Participants also completed the ACQ-R (Anxiety Control Questionnaire-Revised), a 15-item self-report measure of perceived control over aversive events,^4^ the Quick Inventory of Depressive Symptomatology-C^47^ and the Inventory of Depression and Anxiety Symptoms-I.^48^ The Pain Anxiety Symptoms Scale-20, a 20-item self-report measure of fear and anxiety of pain^49^ and the Pain Catastrophizing Scale, a 13-item measure of the tendency to catastrophize during painful situations^50^ also were included. Handedness was assessed using the Edinburgh Handedness Inventory.^51^

### Contextual stimuli

**Figure 1:**
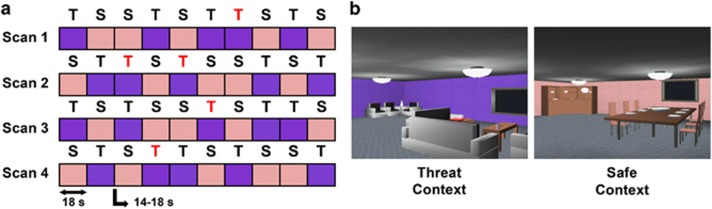
Anticipation of unpredictable threat task. (**a**) During the task, participants explored two contexts, one in which there was a threat of receiving a transcutaneous stimulation at any time (T), and one in which they were safe from receiving any stimulation (S). The acronyms colored in red denote contextual epochs in which unsignaled electrical stimulations were administered, which were limited to one to two threat contexts per run of fMRI scanning. (**b**) Still pictures of the computer-simulated rooms that served as threat and safe contexts. fMRI, functional magnetic resonance imaging.

The stimuli for the scanner task consisted of two computer-simulated rooms generated by a customized software application (Vizard Virtual Reality Toolkit, WorldViz; Santa Barbara, CA, USA; Figure 1). These rooms served as task contexts and were of comparable size and layout but were distinguishable by virtue of their distinctive purple- or peach-colored walls and minor differences in furniture. In one context, participants could receive a transcutaneous electric stimulation on the ankle at any time, whereas in the other context no stimulation could be delivered. Thus, one context was associated with an unpredictable threat and the other context provided safety from threat. During each context presentation, participants could navigate anywhere in the room using a four-button response pad in their dominant hand that allowed them to move forward, backward, left and right in each environment.

### General procedure

Before entering the scanner, participants received task instructions, completed questionnaires and underwent sensor application to allow for nociceptive flexion reflex/pain threshold testing before scanning and the measurement of skin conductance responses (SCRs) during scanning. Following threshold testing, participants practiced using the response pad to navigate the task contexts for 2 min in a full-scale MRI simulator (mock scanner) with no threat of receiving stimulation. Because the present report focuses on SCRs and ratings collected only during task scans, we only briefly describe the overall scanning procedure here. During scanning, participants wore a respiration belt over the abdomen and a nasal cannula at the tip of the nose to monitor breathing, and a finger pulse oximeter to measure heart rate. To minimize unwanted movement in response to electric stimulations, participants wore velcro straps over the hips and left ankle that could be easily fastened and unfastened as needed. Once participants were positioned in the MRI scanner with stabilizing head cushions in place, scanning began with structural and resting-state fMRI scans. Participants then underwent a 4-min practice scan during which they explored the task contexts according to task instructions including receiving one electric stimulation in the threat context. Immediately following the practice scan, participants completed a contingency awareness test in which they confirmed accurate understanding of which context was associated with threat and which was not. The AUT task then commenced.

### AUT task

Before scanning, participants were informed that during the task they were to virtually explore two computer-simulated rooms projected onto a screen in the scanner, and that later they would be asked what they learned about each room. Half the participants were told that whenever they were in the ‘purple room' they could receive a stimulation on the ankle at any time, and that whenever they were in the ‘peach room' they would never receive a stimulation; the other half were given the opposite instructions. During each of the four fMRI scans, five threat and five safe contexts were each semi-randomly presented for a duration of 18 s followed by an interstimulus interval of 14–18 s. Order of scan presentation (for example, 1, 2, 3, 4; 2, 3, 4, 1…) was counterbalanced across participants. An unpredictable (that is, unsignaled) electrical stimulation served as an unconditioned stimulus (US) and was delivered during one to two threat contexts each fMRI scan for a total of five unconditioned stimuli (range 3–16 s post context onset; mean onset=9.6 s). No US was administered during the safe context. During the interstimulus interval, participants performed a low-level vigilance task in which they fixated on a central plus sign and pressed a button on the response pad anytime it changed color (one to two times per interstimulus interval). Following each threat context in which an electric stimulus was administered, participants rated the intensity of the stimulus received on a scale from 0 to 100 presented during the interstimulus interval. Starting from the left, the scale was labeled 0 (no sensation), 25 (uncomfortable), 50 (painful), 75 (very painful) and 100 (maximum tolerable). Participants were allowed 7 s to register an intensity rating.

At the conclusion of each fMRI scan, which lasted 350 s, participants retrospectively rated how fearful they were in the threat and safe contexts using a 0 to 100 scale. Starting from the left, the scale was labeled 0 (no fear), 25 (mild fear), 50 (moderate fear), 75 (strong fear) and 100 (extreme fear). Participants were under no time limit when registering anxiety ratings. Although the AUT task shares similarities with unsignaled contextual fear conditioning, which involves subjects gradually learning context-US associations through direct experience,^13^ the AUT task involves explicit verbal instruction to participants from the outset about which context is associated with threat and which is not. Therefore, it was expected that throughout the task, participants would engage in sustained AUT during the threat context compared with the safe context.

### Electric stimuli and skin conductance assessment

The US during the AUT task was delivered with a Digitimer DS7A (Hertfordshire, UK) constant current stimulator triggered by a presentation computer and waveform generator (Agilent 33220A; Santa Clara, CA, USA). Stimulus intensity was determined before scanning during individual threshold testing. The maximum intensity of stimulation was set at 40 mA. The electric stimulus was delivered via surface electrodes attached to the skin over the left ankle to minimize the amount of head movement during scanning. During each fMRI scan, SCRs were obtained using a Biopac Systems electrodermal activity module. Offline data analysis of SCRs was performed using a model-based general linear model with SCRalyze software (version 2.1.6b; www.scralyze.sourceforge.net).^52, 53^ For details on threshold testing and the assessment of SCRs, see Supplementary Information.

### Data acquisition

Imaging experiments were conducted on a Discovery MR750 3 Tesla MRI scanner (GE Healthcare, Milwaukee, WI, USA). A receive-only 32-element head array coil (Nova Medical, Wilmington, MA, USA) optimized for parallel imaging was used for MRI signal reception. The size and location of the BNST, like other small structures,^54^ make it challenging to study in humans using fMRI.^17^ To address this challenge, we acquired high-resolution functional scans using a single-shot, gradient-recalled echo-planar imaging sequence with sensitivity encoding and following parameters: matrix size: 96 × 96, FOV/slice/gap=240/2.9/0.5 mm, in-plane resolution: 2.5 × 2.5 mm^2^, axial plane: 35 slices, TR/TE=2,000/25 ms, flip angle: 40º, sampling bandwidth: 250 kHz, number of volumes: 175, sensitivity encoding acceleration factor: *R*=2 in the phase-encoding direction. The echo-planar imaging images were reconstructed into a 128 × 128 matrix, in which the resulting voxel size was 1.875 × 1.875 × 2.9 mm^3^. For anatomical reference and alignment purposes, structural MRI scans used a T1-weighted Magnetization Prepared Rapid Gradient Echo imaging sequence with sensitivity encoding (matrix size: 256 × 256, FOV/slice=240/1.1 mm, in-plane resolution: 0.938 × 0.938 mm^2^, TR/TE=5/1.95 ms, inversion and delayed times: TI/TD=725/1,400 ms, flip angle: 8º, sampling bandwidth: 31.25 kHz, number of axial slices per volume: 134, acceleration factor: *R*=2).

### Data preprocessing and subject-level analyses

Functional image preprocessing and analysis was performed using AFNI (http://afni.nimh.nih.gov/afni) and spatial alignment of functional images was performed using Advanced Normalization Tools (http://stnava.github.io/ANTs/) to optimize spatial alignment of functional data to the TT_N27 T1-weighted template (for details, see Supplementary Information). Each subject's data from the AUT task were analyzed using a generalized least squares multiple linear regression model. Functional imaging data at the single subject level were analyzed with 3dREMLfit, a regression program that estimates the serial correlation structure of the noise with an ARMA (1, 1) model, and uses the subsequent temporal correlation matrix to estimate beta parameters using a generalized least squares method. The generalized least squares approach typically produces beta values with smaller variance. The regression model included regressors for each task context as well as regressors of non-interest to account for head motion, signal trends, electric stimulations, stimulation intensity ratings, fixation color changes, end-tidal CO_2_ and navigation behavior. To account for variability of the shape of the BOLD response, each task context was modeled as the sum of piecewise linear B-spline basis functions or tent functions. Fifteen tent functions covering 30 s were used to account for the full extent of each context (0–18 s) and the time span of the BOLD response following context offset. For the contrast of threat context versus safe context, the voxel-wise analysis included only regressors for the 10 time points (0–18 s) spanning each context. The first time point (at 0 s) was assumed to have zero magnitude to account for the expected delay in the BOLD response to context onset. The time points following context offset were treated as regressors of non-interest.

### Group analyses

The behavioral and demographic analyses were carried out using SPSS (IBM SPSS Statistics for Macintosh, Version 22.0, Armonk, NY, USA). Paired-samples *t*-tests were conducted to determine whether SCRs and mean subjective anxiety ratings differed between threat and safe contexts. Independent-samples *t*-tests were conducted to ascertain whether female and male participants differed on demographic and behavioral measures. These statistical tests were two-tailed with *α*=0.05. Following subject-level analyses, a whole-brain voxel-wise analysis was conducted to examine brain BOLD activity during the AUT. This analysis was conducted using AFNI's 3dMEMA program that incorporates individual beta precision estimates into group effects using a mixed-effects meta-analytic approach.^55^ This approach is advantageous because it is robust against the violation of within-subject variability assumptions and some types of outliers, and provides more accurate statistical testing at the group level. The beta values derived from the contrast of threat context versus safe context, which reflect the mean percent signal change during AUT compared with no threat, were extracted for each subject along with the corresponding standard errors. These data were compared across conditions using a one-sample paired *t*-test that included age as a covariate to adjust for subject age in the group analysis. This analysis included only threat trials in which no US was delivered and a comparable number of safe trials. The results of this whole-brain analysis were corrected for multiple comparisons using Monte Carlo simulations. The significance criterion for detecting activation was set at a corrected family-wise error *P*-value of *P*<0.01, determined using the AFNI program 3dClustSim (cluster size ⩾8 voxels), thresholded per voxel at *P*<0.0001. The group sample size was chosen to ensure adequate power to detect effects using these relatively conservative threshold levels.^56^

The STAI-T and ACQ-R were used to conduct correlation analyses examining the relationships among anxiety proneness, perceived control and anterior insula activation during the AUT. For these analyses, the Pearson correlation coefficient was computed using total scores and insula activity was extracted from dorsal (dAI) and ventral (vAI) subregions of anterior insula. More specifically, the average beta values for threat context versus safe context were extracted per individual from dAI and vAI subregions defined by 3-mm-radius spheres placed at the voxel coordinates showing peak activity in the group-level statistical map. The volume of these spheres was selected to balance between preserving the precision of anatomical localization enabled by our high spatial resolution fMRI methods, while also accommodating a modest amount of anatomical variability across subjects. Data from the left and right hemispheres of each subregion were combined to create dAI and vAI subregions, respectively. Therefore, activity in dAI and vAI subregions, respectively, reflect bilateral activation during the AUT.

Additional correlation analyses were performed to explore the relationship between activity in dAI and aMCC and posterior MCC (pMCC) to assess whether aMCC specifically influenced time spent exploring the virtual room in the threat context. The average beta values were derived in the same manner as the data for anterior insula, with aMCC and pMCC activity extracted from subregions defined by 3-mm-radius spheres placed at the voxel coordinates showing peak activity in the group-level statistical map. The average time spent exploring the threat context was measured using the period before any electrical stimulation, and was indexed using the metric total time spent pressing the forward and backward buttons minus total time spent pressing any button (including left and right buttons). Because participants seldom moved in a backward direction, and pressing left and right buttons during navigation would enable participants to orient toward the left and right but not advance forward, it was hypothesized that this metric would best index exploratory or approach behavior during the threat context during AUT. To assess the average time spent exploring overall, in both the threat and safe contexts, the metric used was total time spent pressing the forward and backward buttons minus total time spent pressing any button irrespective of the timing of any electric stimulus in reinforced threat trials.

To examine whether anxiety proneness influenced dAI activation through perceived control, a simple mediation analysis was conducted for dAI. Additional mediation analyses were conducted for vAI, aMCC and the BNST as *post hoc* tests aimed at assessing the specificity of the results in the dAI. These analyses were conducted using ordinary least squares path analysis in the SPSS version of PROCESS^57^ using the identical data used in the correlational analyses. *Post hoc* analyses were performed to examine the relationships between SCR or subjective anxiety with anterior insula and BNST activation. The data for the correlational analyses involving activity in the BNST also were extracted from a 3-mm-radius sphere placed at the peak voxel coordinates in the group-level statistical map.

## Results

On average, participants rated the US intensity as moderately painful (*M*=49, s.d.=15). SCRs and subjective anxiety were greater during threat than safe contexts (SCR: *t*_(39)_=6.24, *P*<0.001; anxiety: *t*_(39)_=7.95, *P*<0.001). No significant difference was observed between female and male participants in anxiety proneness or perceived control, the factors of primary interest (Table 1). They also did not differ significantly in age, body mass index, measures of anxiety and mood symptoms or pain-related negative affect (Table 1). Regardless of normalization of the SCR data, there was no significant difference between females and males in SCRs during the task, anxiety ratings or US intensity ratings (SCR: *t*_(38)_=0.12, *P*=0.91; anxiety: *t*_(38)_=−1.72, *P*=0.09; US: *t*_(38)_=−0.27, *P*=0.78). Thus, the remaining results are based on all participants irrespective of sex.

### Imaging results

**Figure 2:**
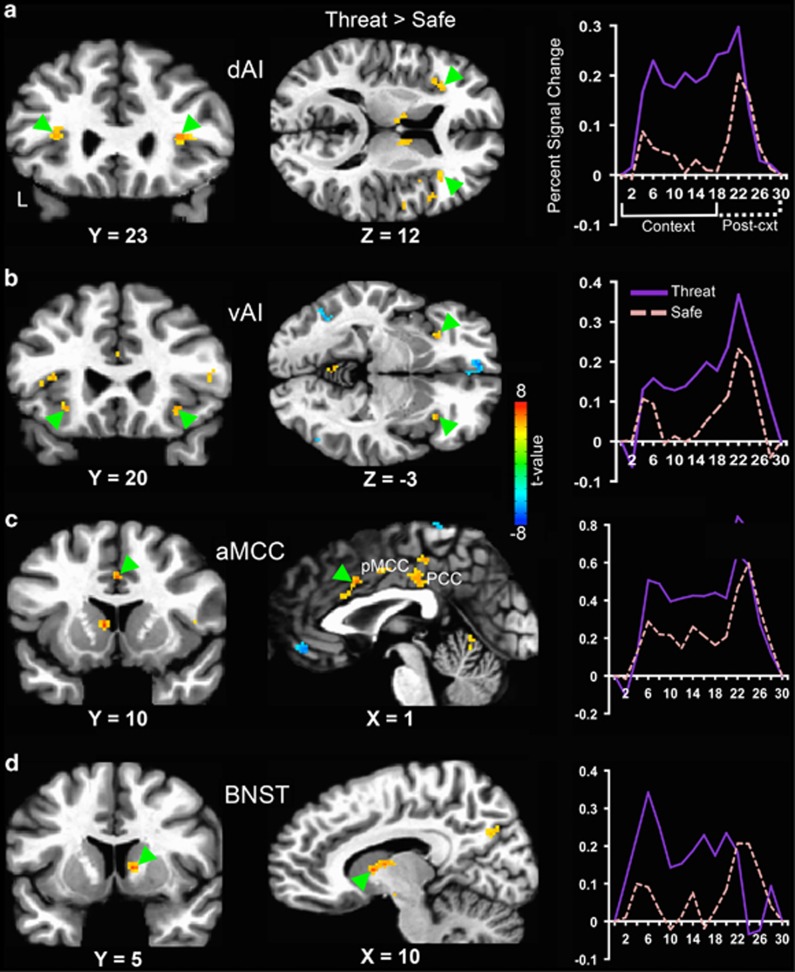
Adult nonclinical subjects exhibited increased hemodynamic activity in (**a** and **b**) the dorsal (dAI) and ventral (vAI) regions of anterior insula, (**c**) anterior midcingulate cortex (aMCC) and (**d**) the bed nucleus of the stria terminalis (BNST; indicated by green triangles) during anticipation of unpredictable threat (threat > safe). To the right are plots depicting the peristimulus time courses of the hemodynamic response in each region. Following a brief delay in the hemodynamic response to context onset, all the four regions showed greater sustained activation during the threat context compared with the safe context. All the results shown were corrected for multiple comparisons at *P*_corr_<0.01. L, left; PCC, posterior cingulate cortex; pMCC, posterior midcingulate cortex.

Relative to the safe context, the mean BOLD signal was significantly greater during the threat context in the dorsal and ventral regions of the anterior insula, aMCC (as well as more posterior subregions of the cingulate cortex) and the BNST (Figure 2; Table 2; also see Supplementary Table S1). The hemodynamic response function indicated that the BOLD signal elevation in these regions was sustained over the entire epoch of the unpredictable threat condition (Figure 2). In addition, several regions exhibited greater activation during the safe context than the threat context including the ventromedial prefrontal cortex and anterior hippocampus (Supplementary Figure S1; Supplementary Table S2).

### Relationships among anxiety proneness, perceived control and anterior insula activation

**Figure 3:**
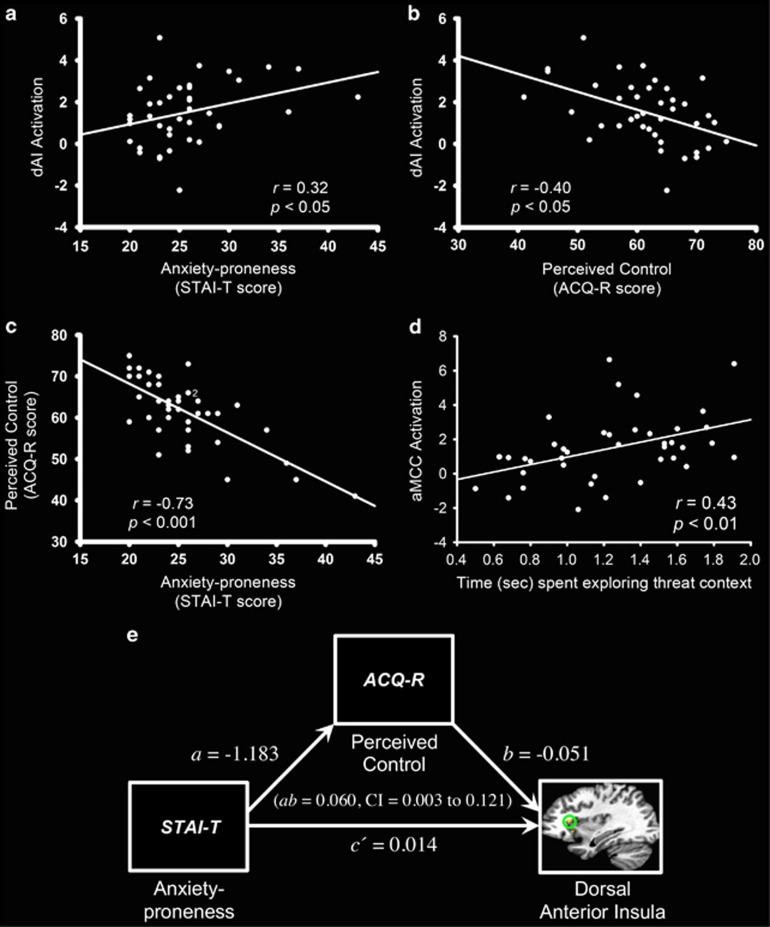
(**a** and **b**) Dorsal anterior insula (dAI) activation is positively correlated with anxiety proneness and negatively correlated with perceived control. (**c**) Anxiety proneness, as measured by trait anxiety scores, is inversely correlated with perceived control over aversive events. The numeral two indicates two overlapping data points. (**d**) Increased activity in anterior midcingulate cortex (aMCC) was associated with greater exploratory behavior in the threat context. Activation values on the y axis are beta coefficients representing mean percent signal change during anticipation of unpredictable threat (threat > safe). (**e**) Schematic representation of the mediation model. The pathway from STAI-T to ACQ-R (path *a*) and then from ACQ-R to dorsal anterior insula (path *b*) represents the indirect effect of anxiety proneness on dAI activity through perceived control (quantified as the product of paths *a* and *b*). The pathway from STAI-T to dAI (path *c*') represents the direct effect of anxiety proneness on dAI activity. Model coefficients are reported in unstandardized form, thus they map directly onto the measurement scales used. A 95% confidence interval (CI) for the indirect effect (*ab*) does not contain and is entirely above zero, thus providing evidence that perceived control serves as a mediator of the effect of anxiety proneness on dAI activity. ACQ-R, Anxiety Control Questionnaire-Revised; STAI-T, Spielberger State-Trait Anxiety Inventory.

The magnitude of the BOLD signal elevation in dAI correlated positively with the extent of anxiety proneness (STAI-T: *r*=0.32, *P*<0.05, Figure 3a) and negatively with perceived control (ACQ-R: *r*=−0.40, *P*<0.05, Figure 3b). In contrast, the corresponding correlations with BOLD activity in the vAI were not significant (STAI-T: *r*=−0.15, *P*=0.36; ACQ-R: *r*=0.20, *P*=0.21). Moreover, the levels of anxiety proneness correlated inversely with perceived control (*r*=−0.73, *P*<0.001, Figure 3c). The magnitude of the BOLD activity change in the dAI correlated positively with that of the aMCC (*r*=0.32, *P*<0.05), but was not significantly correlated with that of the pMCC (*r*=0.29, *P*=0.07). In addition, greater aMCC activation was associated with increased time spent exploring the threat context during AUT (*r*=0.43, *P*<0.01, Figure 3d). By contrast the corresponding correlation with pMCC activation was not significant (*r*=0.24, *P*=0.14). Note that no significant difference was observed between threat and safe contexts in time spent exploring overall (*t*_(39)_=0.14, *P*=0.89, Supplementary Figure S2).

### Mediation analysis results

Mediation analyses were conducted to test whether diminished perceived control may be a mechanism through which anxiety proneness exerts influence specifically on dAI activation. As conveyed in Figure 3e and Supplementary Table S3, individuals more prone to anxiety perceived that they had less control over aversive events (*a*=−1.183), whereas those with lower perceptions of control tended to respond to anticipation of threat with greater activation in dAI (*b*=−0.051). A bias-corrected 95% bootstrap confidence interval (CI) for the indirect effect (*ab*=0.060) based on 10,000 bootstrap samples was entirely above zero (0.003 to 0.121). There was no evidence that anxiety proneness influenced dAI activation independent of its effect on perceived control (*P*=0.787). In contrast, the corresponding analysis with BOLD activity in vAI, aMCC and BNST, respectively, yielded no support for an indirect effect of anxiety proneness on activity in these regions through perceived control (vAI: *ab*=−0.024, CI=−0.082 to 0.031; aMCC: *ab*=−0.053, CI=−0.242 to 0.109; BNST: *ab*=0.001, CI=−0.106 to 0.128; Supplementary Tables S4). Similarly, there was no evidence of a direct effect of anxiety proneness on activity in these regions independent of the influence of perceived control (vAI: *P*=0.986; aMCC: *P*=0.558; BNST: *P*=0.919).

### Relationships between autonomic arousal, subjective anxiety and insula and BNST activation

*Post hoc* correlations suggested multiple relationships between levels of autonomic arousal, subjective anxiety and activation in insula and BNST. Autonomic arousal assessed via SCR was associated with increased subjective anxiety as measured by anxiety ratings (*r*=0.33, *P*<0.05). The change in BOLD activity in the dAI correlated positively with both SCR (*r*=0.32, *P*<0.05) and subjective anxiety (*r*=0.32, *P*<0.05). In contrast, the change in BOLD signal in vAI correlated significantly only with SCR (anxiety: *r*=0.24, *P*=0.14; SCR: *r*=0.41, *P*<0.01). Similarly, the change in BOLD activity in the BNST correlated positively with SCR (*r*=0.34, *P*<0.05), but the relationship between BNST activation and subjective anxiety was not significant (*r*=0.27, *P*=0.10).

## Discussion

We tested the hypothesis that high-trait anxiety may influence the hemodynamic activity of the anterior insula during AUT indirectly through perceived control. The study yielded six major results. First, individuals who showed greater activation in dAI during anticipation of threat relative to no threat, also showed increased levels of trait anxiety and decreased levels of perceived control. Second, individuals with greater levels of trait anxiety also reported lower levels of perceived control, consistent with the extant literature. Third, the magnitude of activation in dAI during anticipation of threat correlated moderately with activation in aMCC. Fourth, greater activation in aMCC during anticipation of threat was related to increased time spent exploring the threat context. Fifth, perceived control mediated the relationship between trait anxiety and activation in dAI during anticipation of threat; moreover, the magnitude of dAI activity correlated positively with the levels of both autonomic arousal and subjective anxiety during the anticipation of unpredictable shock. Sixth, we replicated and extended our previous finding that, in humans, the BNST is activated by sustained anxiety in response to a context associated with unpredictable aversive stimulation,^17^ consistent with translational evidence from studies in experimental animals.^45^ The magnitude of autonomic arousal correlated positively with greater activity in the BNST, as well as in the vAI and dAI. Taken together, these results supported the *a priori* hypothesis and demonstrated an extended functional anatomy of contextual anxiety, which includes the aMCC and the BNST as well as the anterior insula.

Other studies have demonstrated that healthy adults have enhanced activity in insular cortex during the anticipation of aversive events,^7, 8, 9, 10, 11, 12, 13, 14, 15, 16, 17, 18, 19^ and that adult anxiety-prone individuals from a nonclinical sample may have exaggerated anterior insula activity^29, 30, 31^ and altered activity in anterior cingulate during emotional processing.^32^ It has been also demonstrated that there is a large, negative association between trait anxiety and perceived control, such that higher levels of anxiety proneness are related to greater deficits in perceived control.^5^ However, until now, the functional role of hyperactivity in anterior insula in the relationship between elevated trait anxiety and perceived control had not been empirically examined. Our data suggest that the link between anxiety proneness and exaggerated anterior insula activity is mediated by reduced perceived control.

Perceived control is generally characterized by an individual's beliefs about how much control they have and has been implicated in a wide range of life domains.^59, 60^ Over time, according to etiological theories of emotional disorders,^61^ perceived control becomes trait-like and influences anxiety by modulating related brain function. We propose that contexts that signal temporally unpredictable aversive events induce uncertainty about the occurrence of future threat. When individuals face such uncertainty, the anterior insula generates a signal predicting an expected aversive body state that results in increased arousal and anticipatory anxiety, the latter of which appears to involve primarily the dorsal region of anterior insula, potentially because of its greater putative involvement in cognitive evaluative processes. Because dAI is engaged by various forms of cognitive evaluation^62, 63^ and has strong functional connectivity to aMCC,^40^ which is thought to modulate action in response to negative affective information,^33^ we suggest that dAI uses perceptions of control in performing calculations of the likelihood and consequences of anticipated threat. This information is used then to amplify or dampen subjective anxiety, and to influence behavior possibly by relaying threat assessment information to aMCC.

The association between increased BNST activation and autonomic arousal supports the hypothesis that the BNST has a role in modulating sustained anxiety responses,^45^ and suggests that anticipatory responses to unpredictable threat involve multiple processes and their neural substrates.^64^ On the basis of prior studies that have implicated ventromedial prefrontal cortex in responding to safety or pleasure,^65, 66, 67, 68^ and data indicating that ventromedial prefrontal cortex and anterior hippocampus (ventral in rodents) may regulate emotional responses^69^ including the contextual retrieval of conditioned stimuli-no US memory following fear extinction,^70, 71^ we interpret greater activity in these regions in the safe versus threat contexts as contributing to safety processing. However, because anxiety ratings were near zero and there were no independent safety ratings acquired, behavioral correlations to support this interpretation were not obtained.

This study has some limitations. First, the data are cross-sectional, thus, prospective data will be required to definitively separate trait anxiety from current levels of perceived control. Second, although ecologically valid, the measure used to examine behavior under threat reflects relatively small differences in exploratory behavior. Third, although we detected a significant relationship between BNST activation and autonomic arousal, greater statistical power or the use of an online anxiety measure may be needed to detect a similar relationship with reported anxiety.

In conclusion, individuals prone to anxiety show hyperactivity in dAI during anticipation of unpredictable aversive events. Anxiety proneness appears to affect dAI activity indirectly through perceived control, suggesting that the perception of control might contribute to the formation and maintenance of dysregulated anxiety, and may serve as an important treatment target for attenuating insula hyperactivity in anxious individuals.

## Figures and Tables

**Table 1:** Descriptive and inferential statistics for demographic and behavioral measures in study sample

*Variable*	*Females (*n=*20)*		*Males (*n=*20)*		t_*38*_	P
	*Mean*	*s.d.*	*Mean*	*s.d.*		
Age, (years)	31.45	10.81	30.45	9.7	0.308	0.76
BMI (kg/m^2^)	25.22	5.34	26.79	3.3	1.116	0.271
						
*Anxiety proneness*						
STAI-T	25.45	3.65	25.95	6.12	0.314	0.755
						
*Perceived control*						
ACQ-R	61.4	5.6	61.55	10.14	0.058	0.954
						
*Anxiety and mood symptoms*						
STAI-S	24.35	4.64	24	4.34	−0.246	0.807
QIDS-C	1.3	1.3	1.15	1.53	−0.334	0.74
IDAS—depression	26.05	3.78	26.8	5.07	0.53	0.599
						
*Pain-related negative affect*						
PASS-20	12.05	6.78	10.9	10.65	−0.407	0.686
PCS	5.45	4.59	6.55	5.92	0.657	0.515

Abbreviations: BMI, body mass index; ACQ-R, Anxiety Control Questionnaire-Revised; IDAS, Inventory of Depression and Anxiety Symptoms; PASS-20, Pain Anxiety Symptoms Scale—Short Form; PCS, Pain Catastrophizing Scale; QIDS-C, Quick Inventory of Depressive Symptomatology-Clinician Rated; STAI, State-Trait Anxiety Inventory.

**Table 2:** Designated regions of interest exhibiting differences in the hemodynamic response during anticipation of unpredictable threat (threat > safe)

*Hemisphere/location*	*Peak coordinates*^a^			t_*38*_	*No. of voxels*
	*x*	*y*	*z*		
Right BNST	10	5	6	6.88	51
Left dorsal anterior insula	−31	23	12	5.55	35
Right dorsal anterior insula	33	23	12	5.93	26
Anterior midcingulate cortex	1	10	33	6.53	23
Right ventral anterior insula	29	16	−5	6.61	21
Left ventral anterior insula	−25	20	−3	5.51	9

BNST, bed nucleus of the stria terminalis. The x, y, z coordinates indicate distance in millimeters from the anterior commissure in three dimensions: x, right to left; y, anterior to posterior; z, dorsal to ventral with positive values indicating right, anterior or dorsal and negative values left, posterior or ventral, respectively. The number of voxels in each cluster reflects contiguous voxels in which *P*<0.01 after applying appropriate corrections for multiple testing.

a All coordinates reported according to stereotaxic array of Talairach and Tournoux.^58^

## Notes

Dr Drevets is an employee of Janssen Pharmaceutical Companies of Johnson & Johnson. The remaining authors declare no conflict of interest.
