# Supplementary material for: Increased anterior insula activity in anxious individuals is linked to diminished perceived control
Source: Transl Psychiatry. 2015 Jun 30;5(6):e591–. doi: 10.1038/tp.2015.84 (PMC4490294; doi:10.1038/tp.2015.84)
Supplement: Supplementary Information [file tp201584x1.doc]

**Increased anterior insula activity in anxious individuals**

**is linked to diminished perceived control**

Ruben P. Alvarez, Namik Kirlic, Masaya Misaki, Jerzy Bodurka, Jamie Rhudy,

Martin P. Paulus, and Wayne C. Drevets

*Supplemental Information*

**Table of Contents**

Supplemental Methods

1. Threshold Testing and Skin Conductance Assessment

2. Data Preprocessing

Supplemental Tables

1. Table S1: Table of Stereotactic Coordinates for Threat > Safe

2. Table S2: Table of Stereotactic Coordinates for Safe > Threat

3. Table S3: Model Coefficients for Mediation Model of Dorsal Anterior Insula

4. Table S4: Model Coefficients for Mediation Model of Ventral Anterior Insula

5. Table S5: Model Coefficients for Mediation Model of Anterior Midcingulate Cortex

6. Table S6: Model Coefficients for Mediation Model of Bed Nucleus of the Stria Terminalis

Supplemental Figures

1. Figure S1: Ventromedial Cortex and Anterior Hippocampus Activation During the

Safe Context Compared to the Threat Context

2. Figure S2: Graphs Depicting Exploratory Behavior during Threat and Safe Contexts

Supplemental References

**Supplemental Methods**

**Threshold Testing and Skin Conductance Assessment**

Unsignaled electric stimuli were delivered with a Digitimer DS7A constant current stimulator triggered by a presentation computer and Agilent 33220A waveform generator. The stimulation delivered consisted of a train of 5 rectangular pulses of 1 ms duration and 3 ms ISI. Stimulation intensity was set at 1.2 times the level at which both the pain threshold and a nociceptive flexion reflex (NFR) were obtained during threshold testing. The NFR is a lower limb withdrawal response evoked by Aδ fibers, and the threshold for the reflex is significantly correlated with subjective pain . To determine NFR and pain thresholds on an individual basis, two MRI-compatible Ag-AgCl surface electrodes filled with salt-free gel were attached to the left ankle over the retromalleolar pathway of the sural nerve (2 cm posterior to the malleolus) after the skin was cleaned with alcohol and NuPrep to reduce impedance to less than 5kΩ. NFR threshold was assessed using a single ascending series of electric stimulations [^3^](#_ENREF_3), with intensity beginning at 1 mA and increasing in 2 mA steps, with a variable 8-12 ISI, until a reflex was detected and pain threshold determined as explained below. To record the reflex, two Ag-AgCl electrodes (inter-electrode distance 3 cm) were placed over the left biceps femoris muscle 10 cm superior to the popliteal fossa and a ground electrode placed over the left lateral epicondyle of the femur, after cleaning the skin with NuPrep to reduce impedance to less than 10kΩ. A reflex was defined as a mean biceps femoris electromyographic (EMG) response in the 91-150 ms post-stimulus interval that exceeded mean EMG activity during the 60 ms pre-stimulus baseline interval by at least 1.5 standard deviations [^4^](#_ENREF_4). To facilitate relaxation of the leg muscles, participants were lying in a supine position in a MRI simulator during threshold testing.

During threshold testing participants also rated the intensity of each electric stimulus received using a computer-presented scale from 0 to 100 identical to the scale used in the scanner task. As explained to participants prior to threshold testing, pain threshold was defined as the level of stimulation that first feels painful, that is, a rating of 50 on the 0-100 scale. NFR and pain threshold testing proceeded until pain threshold was determined and a reflex was obtained. If pain threshold was reached but no reflex detected, or vice versa, testing continued until both thresholds were met.

Skin conductance recordings were obtained during each fMRI scan with MRI-compatible Ag-AgCl electrodes placed on the medial side of the right foot over the abductor hallucis muscle [^5^](#_ENREF_5), using a Biopac Systems electrodermal activity module. Isolated RF filtered electrode cables were interfaced at a custom MRI patch panel allowing for easy cable access between the magnet room and the control room. Offline data analysis of skin conductance responses was performed using general linear model (model-based convolution analysis of waveforms), utilizing SCRalyze software (version 2.1.6b, available at: www.scralyze.sourceforge.net) to estimate the mean response amplitude for threat and safe conditions, respectively. The skin conductance response (SCR) data were normalized to avoid potential bias due to inter-subject differences in response amplitude originating from differences in skin properties or other peripheral factors. The SCR analysis included only threat trials in which no US was delivered, a comparable number of safe trials, and modeling of all extraneous events including US delivery. Thus, the analysis of SCRs to threat contexts were unbiased by intermittent US delivery.

**Data Preprocessing**

Functional image preprocessing and analysis was performed using AFNI (<http://afni.nimh.nih.gov/afni>). In addition, Advanced Normalization Tools (ANTS; see <http://stnava.github.io/ANTs/>) was used to optimize spatial alignment of functional data to the TT_N27 T1-weighted template. Because ANTs uses symmetric diffeomorphic image registration to perform nonlinear warping on a voxel-by-voxel basis rather than warping all voxels by the same parameters, as is typically done with linear transformation, nonlinear spatial normalization with ANTs can produce more accurate alignment especially at smaller brain structures that may be missed by linear transformation . After discarding the first 5 volumes of each EPI time course to allow the fMRI signal to achieve steady state, slice timing correction was performed using the initial slice acquired at the beginning of each volume as a reference. The structural image was aligned to the first EPI image with align_epi_anat.py script in AFNI. The aligned anatomical image, which was resampled to the same resolution as the EPI image, was warped to the TT_N27 T1-weighted template using ANTs. The template image was resampled to 1.875 mm^3^ voxel size beforehand, which determined the size of the normalized EPI image. After realigning the EPI images to the first volume for motion correction, the EPI images were normalized to the template image using the warping parameters obtained from the anatomy to template alignment. To reduce noise in the images without losing substantial spatial resolution, the EPI data were spatially smoothed with a small 1.875 mm FWHM Gaussian kernel. The signal intensity of each EPI volume was then normalized so as to reflect percent signal change from the mean intensity of each voxel across the time course.

**Table S1.** Brain regions exhibiting differences in the hemodynamic response during anticipation of unpredictable threat (Threat > Safe) from the whole brain voxel-wise analysis including areas situated outside the preselected regions of interest (which had been delimited to test the a priori hypotheses).

Peak Coordinates*^a^* # of

Hemisphere/Location x y z t_38_ Voxels

R parietal operculum 52 -31 21 7.49 81

Posterior cingulate cortex -1 -25 36 5.87 65

**R bed nucleus of the stria terminalis** 10 5 6 6.88 51

R inferior frontal gyrus 40 18 14 5.51 44

Midline thalamus -3 -7 6 6.48 43

**L dorsal anterior insula** -31 23 12 5.55 35

R precuneus 10 -67 29 5.06 27

**R dorsal anterior insula** 33 23 12 5.93 26

Cerebellum -1 -55 -1 5.83 23

**Anterior midcingulate cortex** 1 10 33 6.53 23

L precuneus -8 -74 34 5.49 23

**R ventral anterior insula** 29 16 -5 6.61 21

R posterior midcingulate cortex 3 -3 38 5.66 21

L medial caudate -7 10 6 7.23 18

R inferior parietal 48 -46 31 5.80 16

R anteromedial insula/frontal operculum 37 8 10 5.26 14

Posterior cingulate cortex 1 -29 46 5.60 14

R lateral hypothalamic area/substantia nigra 7 -8 -11 7.28 13

R precentral gyrus 53 -3 10 5.39 13

R superior temporal gyrus 55 -50 31 4.85 12

R superior frontal gyrus, lateral 25 40 19 6.95 10

Posterior cingulate cortex 7 -42 23 6.00 10

**L ventral anterior insula** -25 20 -3 5.51 9

R superior frontal gyrus, medial 5 35 32 5.20 9

L, left; R, right. *^a^*All coordinates reported according to stereotaxic array of Talairach and Tournoux [^8^](#_ENREF_8). The x, y, z coordinates indicate distance in millimeters from the anterior commissure in three dimensions: x, right-to-left; y, anterior-to-posterior; z, dorsal-to-ventral with positive values indicating right, anterior, or dorsal and negative values left, posterior, or ventral, respectively. The number of voxels in each cluster reflects contiguous voxels in which p<.01 after applying appropriate corrections for multiple testing. The regions of primary interest highlighted in the main text are shown here bolded.

**Table S2.** Brain regions exhibiting differences in the hemodynamic response for Safe > Threat from the whole brain voxel-wise analysis including areas situated outside the regions highlighted in the main text.

Peak Coordinates*^a^* # of

Hemisphere/Location x y z t_38_ Voxels

L postcentral gyrus -57 -18 33 6.73 158

**L ventromedial prefrontal cortex** -1 40 -7 7.83 104

L paracentral lobule -5 -33 64 6.40 59

R postcentral gyrus 7 -38 68 6.58 50

L posteromedial insula -33 -10 14 6.19 44

L middle temporal -40 -61 -1 6.44 33

R middle temporal 48 -65 -5 6.59 30

L angular gyrus -27 -68 44 6.60 27

R fusiform gyrus 42 -37 -22 5.70 23

R fusiform gyrus 38 -52 -16 5.32 22

L inferior parietal -46 -29 42 5.67 22

**L anterior hippocampus** -18 -10 -20 5.77 19

R parahippocampal gyrus 25 -29 -14 5.61 19

R precentral gyrus 50 -8 32 5.22 18

**R anterior hippocampus** 18 -7 -16 5.74 18

R precentral gyrus 44 -10 31 5.66 17

R postcentral gyrus 57 -14 38 5.70 14

L paracentral lobule -7 -27 66 6.13 9

L parahippocampal gyrus -23 -29 -14 5.37 8

R inferior parietal 44 -35 44 4.91 8

L, left; R, right. *^a^*All coordinates reported according to stereotaxic array of Talairach and Tournoux [^8^](#_ENREF_8). The x, y, z coordinates indicate distance in millimeters from the anterior commissure in three dimensions: x, right-to-left; y, anterior-to-posterior; z, dorsal-to-ventral with positive values indicating right, anterior, or dorsal and negative values left, posterior, or ventral, respectively The number of voxels in each cluster reflects contiguous voxels in which p<.01 after applying appropriate corrections for multiple testing. Regions highlighted in the main text are shown here bolded.

**Table S3.** Model coefficients for the simple mediation model of the dorsal anterior insula (dAI)

Consequent

*M* (Perceived Control) *Y* (Dorsal Anterior Insula)

Antecedent Coeff. SE *p* Coeff. SE *p*

*X* (Anxiety-Proneness) *a* -1.183 0.181 < .001 *c***´** 0.014 0.051 .787

*M* (Perceived Control) *b* -0.051 0.032 .115

Constant *i_1_* 91.879 4.725 < .001 *i_2_* 4.107 3.041 .185

*R^2^* = 0.530 *R^2^* = 0.160

*F*(1, 38) = 42.925, p < .001 *F*(2, 37) = 3.536, p < .05

**Table S4.** Model coefficients for the mediation model of the ventral anterior insula (vAI)

Consequent

*M* (Perceived Control) *Y* (Ventral Anterior Insula)

Antecedent Coeff. SE *p* Coeff. SE *p*

*X* (Anxiety-Proneness) *a* -1.183 0.181 < .001 *c***´** -0.001 0.039 .986

*M* (Perceived Control) *b* 0.020 0.024 .405

Constant *i_1_* 91.879 4.725 < .001 *i_2_* -0.426 2.338 .856

*R^2^* = 0.530 *R^2^* = 0.041

*F*(1, 38) = 42.925, *p* < .001 *F*(2, 37) = 0.781, *p* = .466

**Table S5.** Model coefficients for the mediation model of the anterior midcingulate cortex (aMCC)

Consequent

*M* (Perceived Control) *Y* (Right aMCC)

Antecedent Coeff. SE *p* Coeff. SE *p*

*X* (Anxiety-Proneness) *a* -1.183 0.181 < .001 *c***´** 0.055 0.093 .558

*M* (Perceived Control) *b* 0.045 0.057 .435

Constant *i_1_* 91.879 4.725 < .001 *i_2_* -2.709 5.509 .627

*R^2^* = 0.530 *R^2^* = 0.017

*F*(1, 38) = 42.925, *p* < .001 *F*(2, 37) = 0.311, *p* = .734

**Table S6.** Model coefficients for the mediation model of the bed nucleus of the stria terminalis (BNST)

Consequent

*M* (Perceived Control) *Y* (Right BNST)

Antecedent Coeff. SE *p* Coeff. SE *p*

*X* (Anxiety-Proneness) *a* -1.183 0.181 < .001 *c***´** -0.008 0.079 .919

*M* (Perceived Control) *b* -0.001 0.049 .984

Constant *i_1_* 91.879 4.725 < .001 *i_2_* 1.776 4.678 .706

*R^2^* = 0.530 *R^2^* = 0.001

*F*(1, 38) = 42.925, *p* < .001 *F*(2, 37) = 0.009, *p* = .992


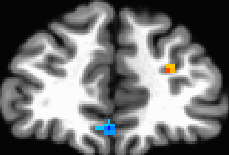


Y = 40


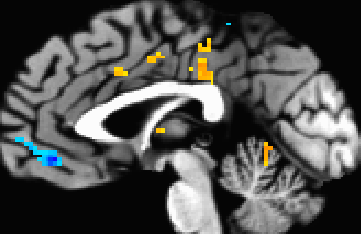


X = -1

-8

8

t-value

*p _corr_* < 0.01


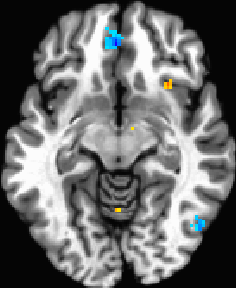


Z = -7

Safe > Threat


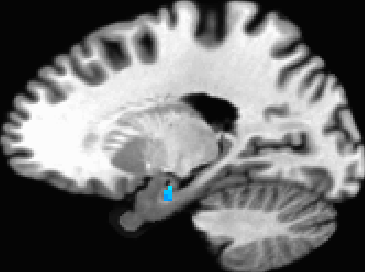


**L**


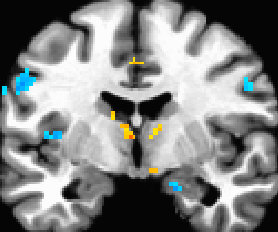


**R**

Y = -7

vmPFC

aHIPP

X = -18

**Figure S1.** Brain images of ventromedial prefrontal cortex (vmPFC, peak voxel coordinates: x=-1, y=40, z=-7) and left and right anterior hippocampus (aHIPP, peak voxel coordinates: L, x=-18, y=-10, z=-20; R, x=18, y=-7, z=-16) exhibiting a greater hemodynamic response to the safe context compared to the threat context. All results shown were corrected for multiple comparisons at *p _corr_* < 0.01. L, left; R, right.


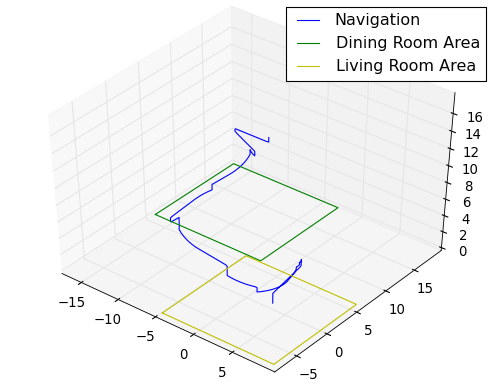

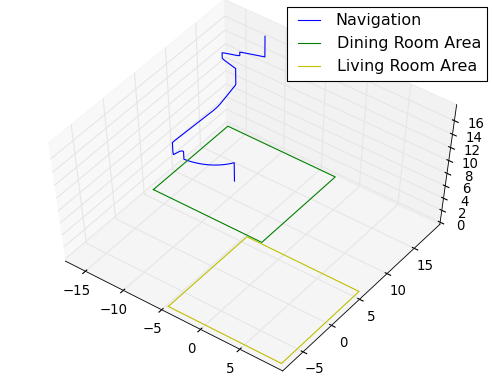


**Threat Context**

**Safe Context**

**Figure S2.** Graphs depicting the basic navigation behavior for a typical subject during a representative trial in the threat context (left) and safe context (right). In this example, the subject can receive a shock at any time in the living room area (i.e., purple room) and is safe from shock in the dining room area (i.e., peach room). Viewed from the top, the vertical axis represents time (t), and the yellow and green boxes at t=0 show the room boundaries of the threat and safe contexts, respectively. Subjects navigated only one room during each trial lasting 18 seconds. As noted in the main text, no significant difference was observed between threat and safe contexts in time spent exploring overall (*t*_(39)_=0.14, *p*=0.89).

**Supplemental References**

1. Chan C, Dallaire M. Subjective pain sensation is linearly correlated with the flexion reflex in man. *Brain Research* 1989; **479:** 145-150.

2. Willer J. Comparative study of perceived pain and nociceptive flexion reflex in man. *Pain* 1977; **3:** 69-80.

3. Rhudy JL, France CR. Reliability and validity of a brief method to assess nociceptive flexion reflex (NFR) threshold. *The Journal of Pain* 2011; **12**(7)**:** 782-791.

4. Rhudy JL, France CR. Defining the nociceptive flexion reflex (NFR) threshold in human participants: a comparison of different scoring criteria. *Pain* 2007; **128**(3)**:** 244-253.

5. Fowles DC, Christie MJ, Edelberg R, Grings WW, Lykken DT, Venables PH. Publication recommendations for electrodermal measurements. *Psychophysiology* 1981; **18**(3)**:** 232-239.

6. Avants B, Epstein CL, Grossman M, Gee JC. Symmetric diffeomorphic image registration with cross-correlation: evaluating automated labeling of elderly and neurodegenerative brain. *Medical Image Analysis* 2008; **12**(1)**:** 26-41.

7. Klein A, Andersson J, Ardekani BA, Ashburner J, Avants B, Chiang M-C *et al.* Evaluation of 14 nonlinear deformation algorithms applied to human brain MRI registration. *NeuroImage* 2009; **46:** 786-802.

8. Talairach J, Tournoux P. *Co-planar Stereotaxic Atlas of the Human Brain*. Thieme Medical Publishers: New York, 1988.
